# Autoimmune bullous diseases during COVID-19 pandemic: 2022 update on rituximab and vaccine

**DOI:** 10.3389/fmed.2023.1112823

**Published:** 2023-01-19

**Authors:** Anna Pira, Jo Linda Maria Sinagra, Francesco Moro, Feliciana Mariotti, Giovanni Di Zenzo

**Affiliations:** ^1^Molecular and Cell Biology Laboratory, Istituto Dermopatico dell’Immacolata (IDI)-IRCCS, Rome, Italy; ^2^Dermatology Unit, Istituto Dermopatico dell’Immacolata (IDI)-IRCCS, Rome, Italy

**Keywords:** COVID-19, SARS-CoV-2, autoimmune bullous diseases, bullous pemphigoid, pemphigus, vaccine, rituximab

## Abstract

Autoimmune bullous diseases (AIBDs) are a heterogeneous group of life-threatening disorders associated with subepidermal or intraepidermal blistering. Skin barrier alterations and prolonged immunosuppressive treatments increase the risk of infections in patients with AIBDs, who are considered fragile. COVID-19 pandemic had a heavy impact on these patients. Although advances have been made in terms of prevention and treatment of COVID-19, this topic remains significant as the pandemic and its waves could last several years and, so far, a relevant proportion of the population worldwide is not vaccinated. This review is a 2022 update that summarizes and discusses the pandemic’s burden on AIBD patients mainly considering relevant studies in terms of: (i) sample dimension; (ii) quality of control populations; (iii) possible standardization by age, gender and country. The findings show that: (i) the risk of COVID-19 infection and its severe course were comparable in AIBD patients and in the general population, except for rituximab-treated patients that presented a higher risk of infection and severe disease; (ii) the mortality rate in COVID-19-infected bullous pemphigoid patients was higher than in the general population, (iii) 121 cases of AIBD onset and 185 cases of relapse or exacerbation occurred after COVID-19 vaccination and a causal relationship has not been demonstrated so far. Altogether, acquired knowledge on COVID-19 pandemic could also be important in possible, albeit undesirable, future pandemic scenarios.

## Introduction

Since December 2019 an outbreak of the novel coronavirus (SARS-CoV-2) disease has quickly spread worldwide. The infectious disease and pneumonia caused by this virus is called 2019 coronavirus diseases (COVID-19). Globally, as of 25 November 2022, there have been 636,440,663 confirmed cases of COVID-19 (7.96% of the world population), including 6,606,624 deaths (1.04% of confirmed cases), reported to the World Health Organization (1). Hospitalization is needed for 3–20% of COVID-19 patients; among these, 10–30% require intensive care (2, 3). Age, obesity, male sex, and comorbidities such as hypertension, heart disease, diabetes, kidney failure, and chronic pulmonary disease are considered as risk factors (4–10). Autoimmune bullous diseases (AIBDs) are a heterogeneous group of disorders including bullous pemphigoid (BP), mucous membrane pemphigoid (MMP), pemphigus, pemphigoid gestationis (PG), linear IgA bullous dermatosis (LABD). These disorders are associated with subepidermal or intraepidermal blistering and their treatment usually involves specific immunosuppressive therapies. Due to skin barrier alterations and chronic immunosuppressive treatments, patients are potentially at greater risk of infections, and they are considered fragile.

This review aims to discuss the pandemic’s burden on AIBD patients in terms of (i) susceptibility to COVID-19 infection; (ii) the relationship between infection and disease course and treatment; (iii) possible effects of vaccination in terms of induction, exacerbation or relapse of autoimmune disease.

## Methods

To analyze the prevalence of COVID-19 in AIBD patients, and the effect exerted by immunosuppressive treatments in AIBD patients we qualitatively selected studies based on: (i) sample size; (ii) quality of control populations; (iii) standardization by age, gender, and country. To illustrate the spectrum of reported cases of new onset, relapse and/or worsening of AIBDs after COVID-19 vaccination all published cases found in PubMed searches up to November 2022 have been included in the present review.

## Prevalence of COVID-19 in AIBDs patients

A recent systematic review analyzed the proportion of AIBD patients with COVID-19 symptoms and positive molecular test (11). This review included 732 subjects affected by AIBDs (409 not specified, 211 pemphigus, 112 pemphigoid). Almost all information was collected *via* phone/telemedicine visits. COVID-19 symptoms were reported in 70 (9.5%) patients, and the diagnosis was confirmed in 16 (2.2%) patients (11). At present, 11 relevant studies reported prevalence data on SARS-CoV-2 infection in AIBD patients, with a pooled total of 10,060 patients (Table 1). COVID-19 symptoms were observed in 256 patients (2.5%), of whom 162 (1.6%) had a confirmed diagnosis (12–22) (Table 1). In order to compare prevalence data between AIBD patients and the general population, two recent studies performed a standardization by age, gender, country, or region of interest and period involved (12, 13). Kridin et al. carried out a retrospective cohort study on 3,081 patients with BP and pemphigus from the data set of Clalit Health Services in Israel. The adjusted hazard ratio (HR) between confirmed COVID-19 patients and controls was 1.1 [95% confidence interval (CI):0.71–1.71] for BP, 0.79 (95% CI: 0.43–1.46) for pemphigus (13). In parallel, 49 dermatology departments in France enrolled 5,180 AIBD patients and found that 29 (0.6%) had a confirmed COVID-19 diagnosis while 59 (1.1%) had possible, probable, or confirmed COVID-19 diagnosis. The standardized incidence ratio of hospitalized confirmed COVID-19 infection was 0.42 (95% CI: 0.20–0.80) for BP, 1.02 (95% CI: 0.37–2.26) for pemphigus, and 1.18 (95% CI: 0.55–2.23) for MMP (12). Altogether, these studies confirmed that the risk of COVID-19 infection in AIBDs patients was not higher than in the general population.

**TABLE 1:** Summary of relevant published studies reporting prevalence data on SARS-CoV-2 infection in AIBDs patients.

References	No. of pts	Diagnosis	SARS-CoV-2 positive pts	Diagnosis of positive pts	% of positive pts, confirmed only	% of positive pts, confirmed/ Possible/Probable
(19)	93	62 BP; 31 PV	5 confirmed 12 possible/probable	Confirmed: 4 BP, 1 PV; Possible/probable: 6 BP, 6 PV	5.38%	18.20%
(16)	43	30 BP; 9 pemphigus; 4 MMP	1 confirmed	Pemphigus	2.32%	2.32%
(17)	83	Unspecified AIBDs	1 confirmed 17 possible/probable	Unspecified AIBDs	1.20%	21.69%
(20)	167	PV	5 confirmed	PV	2.99%	2.99%
(22)	48	Pemphigus	1 confirmed	Pemphigus	2.08%	2.08%
(14)	704	620 pemphigus; 54 BP; 24 MMP; 3 linear IgA disease; 2 EBA; 1 PG	21 confirmed 35 possible/probable	Unspecified AIBDs	2.98%	7.95%
(13)	3,081	1,845 BP; 1,236 pemphigus	36 confirmed	24 BP, 12 pemphigus	1.17%	1.17%
(15)	383	11 unspecified AIBD; 207 PV; 75 MMP; 59 BP; 31 PF	11 confirmed	Unspecified AIBDs	2.90%	2.90%
(18)	31	Pemphigus	1 confirmed	PF	3.20%	
(12)	5,180	Unspecified AIBDs	29 confirmed 30 possible/probable	Confirmed: 9 BP, 13 MMP, 6 pemphigus, 1 PG; Possible/probable: unspecified AIBDs	0.56%	1.10%
(21)	247	Pemphigus	51 confirmed	Pemphigus	20.60%	20.60%

AIBD, autoimmune bullous disease; BP, bullous pemphigoid; EBA, epidermolysis bullosa acquisita; MMP, mucous membrane pemphigoid; PF, pemphigus foliaceus; PG, pemphigoid gestationis; PV, pemphigus vulgaris; Pts, patients.

Although AIBD patients receiving rituximab (RTX) are more prone to viral infections than the normal population (23), only few studies investigated the possible influence of RTX-induced B cell depletion on the SARS-CoV-2 infection rate. A cohort study on 704 AIBD patients reported that among 21 COVID-19 confirmed cases, 14 (66.7%) received RTX within the last 12 months. Interestingly, the relative risk (RR) of COVID-19 infection and hospitalization decreased by 38% (95% CI: 18–57%) and 45% (95% CI: 15–72%), respectively, with each passing month from the last RTX infusion (14). In line with this observation, Joly and French Study Group on Auto Immune Bullous Skin Diseases, and the French Network of Rare Diseases in Dermatology [FIMARAD] reported an incidence ratio of COVID-19 in 23 RTX-treated patients ranging from 3.62 (95% CI: 1.29–8.85) in hospitalized patients with a confirmed diagnosis to 5.37 (95% CI: 3.15–8.96) in patients with a confirmed, probable, or possible diagnosis of COVID-19 (12). On the other hand, Breglio et al. found no difference in the rate of positive SARS-CoV-2 tests between 11 tests collected from RTX-treated patients and 66 tests collected from patients that were either never treated with RTX or received the last infusion at least 1 year before the onset of the pandemic (9.1 vs. 12.1%). However, the number of positive tests does not necessarily match the number of positive patients, since one patient could have been tested multiple times. In addition, the incidence could have been overestimated because patients performing SARS-CoV-2 test are usually symptomatic (24). On the contrary, Özgen et al. reported that among 51 of 247 pemphigus patients with COVID-19, 33% were treated with RTX while among non-COVID-19 patients only 21% were RTX-treated. These findings once more suggested that RTX increases the risk of COVID-19 (21). Of note, among AIBD patients, 45% of SARS-CoV-2 infected and 78% of non-infected were vaccinated; interestingly, only 29% of infected and RTX-treated patients were vaccinated (21), suggesting a role of the vaccine in preventing infection.

Thus, even though these results should be interpreted with some caution due to their relatively small sample size, the risk of SARS-CoV-2 infection seemed higher in RTX-treated patients than in those who did not receive a B cell depletion therapy.

## The influence of AIBD and its immunosuppressive treatment on COVID-19 clinical course and vice versa

Two recent studies investigated the risk of severe COVID-19 outcomes in patients with immune-mediated inflammatory diseases (25, 26), reporting a similar risk of COVID-19-related death and hospitalization in patients and the general population. In addition, some evidence suggests that immunocompromised patients do not have a higher incidence or complications from COVID-19 than the general population (27, 28). In line with these studies, a systematic review suggested that immunomodulatory treatments of AIBDs do not increase the risks of poor outcomes in COVID-19 patients (11). Later, other studies investigated the course of COVID-19 in AIBD patients. Özgen in Turkey enrolled 51 patients having COVID-19 of whom 40 (78%) had a non-serious disease, 11 (22%) required hospitalization, and one (2%) died (21). In Iran Mahmoudi et al. followed 21 AIBD patients with confirmed COVID-19 diagnosis: 15 (71%) had been hospitalized, 7 (33%) needed intensive care facilities and 3 (14%) died. Of note, two studies found that COVID-19 infection and hospitalization rates were higher with prednisolone dosages > 10 mg/die (14, 29). In parallel, Kridin et al. in Israel followed 36 confirmed COVID-19 cases in a cohort of 3,081 AIBD patients, of whom 17 (47.2%) were hospitalized, and 7 (19.4%) died (13). Similar results were observed in a larger French cohort of 5,180 AIBD patients where Joly and French Study Group on Auto Immune Bullous Skin Diseases, and the French Network of Rare Diseases in Dermatology [FIMARAD] identified 59 possible, probable, or confirmed COVID-19 cases of whom 30 (50.8%) were hospitalized, 7 (11.9%) were admitted to an intensive care unit, and 15 (25.4%) died (12). The latter two studies on the largest cohorts analyzed indicated that the risk of hospitalization was comparable to the general population (13) and that exposure to systemic corticosteroids and immunosuppressive agents was not significantly associated with increased severity of COVID-19 (12, 13). However, both studies reported a higher mortality rate for BP patients. In particular, Kridin et al. reported that COVID-19-associated mortality was higher in BP patients (adjusted HR 2.81) compared to the control population, while in pemphigus patients it was similar (adjusted HR 1.15) to their controls (13). Similar results are stated in Joly and French Study Group on Auto Immune Bullous Skin Diseases, and the French Network of Rare Diseases in Dermatology [FIMARAD]’s study, where mortality risk in confirmed COVID-19 patients was 1.63-fold higher in hospitalized patients with AIBDs than in the aged-matched population (12).

### RTX could affect the COVID-19 disease course

A major concern in management of AIBDs during the pandemic is the safety of RTX treatment. In patients with rheumatologic disease, RTX treatment increased the hospitalization and mortality rate and led to an unfavorable disease course (30–35). Recently, a study on a very large cohort of patients affected by immune-mediated inflammatory diseases found that RTX therapy was associated with increased COVID-19-related death (HR 1.68, 95% CI: 1.11–2.56) (25). To evaluate the effect of RTX treatment on the COVID-19 disease course several studies were conducted also on AIBD patients. Few studies assessed a favorable COVID-19 disease course in RTX-treated patients (20, 22). However, none of the reported patients were infected close to the time of RTX infusion, when B cell depletion was profound. In this context, Özgen et al. on a cohort of 51 patients, found a higher percentage of hospitalization in RTX-treated vs. non-treated patients (33.35 vs. 23.52%), but not a higher mortality (21). In line with this study, Mahmoudi et al. found that the RR of being infected with COVID-19 and being hospitalized decreased by 38% (95% CI: 18–57%) and 45% (95% CI: 15–72%), respectively, with each passing month from the last RTX infusion (14). Hwang and Tomayko reported that among 19 patients with AIBDs and COVID-19 infection two patients, treated with RTX < 5 months before, deceased. However, in both cases, they were elderly (74 and 82 years old) and affected by several comorbidities (36). In our experience, recent treatment with RTX could correlate with a more severe course, but not necessarily with an unfavorable prognosis (18). Most of these studies involved unvaccinated patients, but COVID-19 course could be less severe in vaccinated patients. In fact, circulating anti-microbial antibodies appear to arise from long-lived plasma cells. RTX does not abrogate these cells, as they are not dependent on CD20 positive memory B cells, such as reported in mice where maintenance of the plasma cell pool is independent of memory B cells (37). A recent study from Moghadam et al. evaluated 93 AIBD patients with a confirmed diagnosis of COVID-19. Interestingly, the authors found that vaccination and each passing month from the last RTX dose attenuated disease severity (29).

### Disease worsening or induction following SARS-CoV-2 infection

There is evidence that SARS-CoV-2 could hyper-stimulate the immune system inducing autoantibodies’ synthesis, and possibly triggering autoimmune diseases (38). Despite this, very few cases of COVID-19-induced AIBDs have been published (39). More frequently, COVID-19 infection seems to trigger AIBD worsening/flare. Ghalamkarpour and Pourani described a case of pemphigus vulgaris (PV) flare after COVID-19 infection (40). According to Özgen et al. among 51 patients 10 (19.6%) experienced disease flare after COVID-19, and five of them (50%) needed further treatment or therapy adjustment due to pemphigus flare (21). In accordance, Kasperkiewicz et al. found a moderate to severe AIBD worsening in 35.2% of patients through a web-based survey (15). Recently, Moghadam et al. reported that 18 out of 93 (19.3%) AIBD patients experienced a disease flare following COVID-19 infection (29).

## New-onset of AIBDs associated with COVID-19 vaccines

Several case reports, case series, and a recent Italian multicenter study proposed a possible, so far unproven, causal relationship between COVID-19 vaccination and AIBD onset (Supplementary Table 1) (41–91). To our knowledge 51 studies reporting new-onset AIBDs, with a total of 121 patients that developed BP (92 cases), anti-p200 pemphigoid (1 case), pemphigus (1 case), PV (15 cases), pemphigus foliaceus (PF) (7 cases), pemphigus vegetans (1 case), and LABD (4 cases) following SARS-CoV-2 vaccination (Supplementary Table 1) were published. Information about age and sex was not reported for 17 patients. The remaining cases included 58 men and 46 women (M/F = 1.3), ranging from 23 to 97 years (median age: 76 years). Information about administered vaccines was not reported for five patients, and latency to lesions was not reported for 17 patients. According to its greater employment, AIBD onset was more frequently associated with the Pfizer vaccine (61.2%). The onset of AIBDs followed the first dose in 44 patients, the second dose in 47 patients, and the third dose in 13 patients; median latency time was 9, 7, and 14 days from the first, second, and third dose, respectively. Information about the diagnosis was not reported for eight patients. The remaining 113 subjects had a diagnosis confirmed by consistent histopathological and/or serological findings. All patients were treated with corticosteroids and/or immunosuppressive drugs, and the vast majority showed a good clinical response (Supplementary Table 1). The largest cohort belongs to an Italian multicenter study that enrolled 21 patients with COVID-19 vaccine-associated BP (65). The authors concluded that BP patients did not differ essentially from idiopathic BP; however, a male predominance and a reduced autoantibody response to BP230 were considered unique features of vaccinated BP cases. Of note, a recent study by Birabaharan et al. on a very large cohort of individuals found no difference in the risk of BP onset among persons receiving the mRNA COVID-19 vaccine compared to an unvaccinated matched control cohort (92), suggesting that in a period with global mass vaccinations, the association between AIBDs occurrence and vaccine could be a random coincidence. A limitation of the study is the only use of International Classification of Diseases (ICD) codes from database sources for BP diagnosis.

A possible speculation that may bring to an agreement among the real-life clinical observations on patients with a new-onset of an AIBD and the convincing data from Birabaharan et al. considers the vaccination as a precipitating factor. Specifically, the vaccine may induce autoimmunity in genetically predisposed individuals by stimulating a pre-existent, and sub-clinical autoreactivity against hemidesmosomal components. This phenomenon could slightly anticipate BP development without significantly modifying the incidence of the disease. Additional investigations on this controversial topic are needed.

## Relapses and worsening of AIBD after SARS-CoV-2 vaccination

Several vaccinations such as tetanus and influenza have been reported to exacerbate AIBDs (93, 94). The mechanism mentioned above for AIBD onset after vaccination could also be considered for vaccine-associated relapse or worsening of a pre-existing disease. In fact, even in patients who achieve disease control or remission, clinical or sub-clinical autoreactivity against hemidesmosomal components is probably present. Kasperkiewicz and Woodley analyzed 30 published papers on AIBD during the pandemic and reported that 10% of patients had a flare or worsening following vaccination (95). To date, relapse/worsening after COVID-19 vaccine has been described in 185 patients (21, 42, 55, 61, 69, 75, 79, 82, 83, 96–100) (Table 2). Considering cases with known gender and age, the M/F ratio was 0.8 (8 M, 10 F). Ages ranged from 28 to 83 years, and the median age was 62 years. Similar to new-onsets, relapse and worsening were more frequently associated with the Pfizer vaccine (44.4%), as expected. The relapse/worsening of AIBDs followed the first dose in 27 patients, the second dose in 10 patients and the third dose in three patients; mean latency time was 7.5, 11.2, and 15.1 days from the first, second, and third dose, respectively (Table 2). Most patients were treated with corticosteroids and/or immunosuppressive drugs and showed a good clinical response (Table 2). Of note, it should be considered that exacerbation of the disease after the first dose does not preclude the administration of a second dose. In fact, very few patients presented disease exacerbation after both doses (83, 96) (Table 2). In general, disease relapse/worsening events can be reduced if the AIBD is stable at the time of vaccination (83). Of note, the risk of exacerbation should not dissuade from getting the vaccine.

**TABLE 2:** Demographics, vaccines, latency time, and clinical features of AIBD patients reporting disease flare or worsening after COVID-19 vaccination.

References	Pts	Sex, age (y)	SARS-CoV-2 vaccine	Latency to lesions	Therapy	Outcome
(96)	3 BP	F, 74	CV	7 d after I dose	SCS	NA
		F, 65	CV	7 d after II dose	SCS	NA
		M, 71	CV	45 d after II dose	SCS	NA
(42)	1 BP	M, 83	P	5 d after I dose	SCS, TCS	Ongoing at d45
(61)	3 BP	F, 74	CV	7 d after I dose	TCS, SCS, DCN, MTX	Improvement
		F, 65	CV	7 d after II dose	TCS, MTX	Improvement
		M, 71	CV	45 d after II dose	TCS, SCS, AZA	Improvement
(75)	2 BP	F, 57	Mod	7 d after III dose	TCS	Complete remission
		M, 62	P	7 d after III dose	TCS, SCS	Complete remission
(82)	1 BP	M, 54	P	14 d after I, II, and III dose	TCS	Remission
(83)	1 BP	NA	NA	2 d after I dose	TCS	NA
(96)	2 PV	M, 40	Mod	3 d after I dose	SCS, MMF	NA
		M, 80	P	3 d after I dose	SCS	NA
(55)	1 PV	M, 62	AZ	7 d after I dose	SCS	NA
(97)	1 PV	F, 35	CV	5 d after II dose	SCS, RTX	Remission
(69)	2 PV	F, 58	CV	within 7 d after I and II dose	SCS, Ig	Partial remission
		F, 31	P	7 d after I dose	SCS	Complete remission
(21)	18 pemphigus	NA	38.9% CV, 61.1% P	83.3% after I dose, 16.7% after II dose	NA	NA
(98)	1 PV	F, 46	Mod	7 d after I dose	SCS, RTX	Improvement
(99)	3 pemphigus	NA	NA	7, 14, and 18 d after I, II, or III dose, respectively (mean values)	NA	NA
(79)	1 PV	F, 28	CV	14 d after I dose	SCS, RTX	Improvement
(83)	58 PV	NA	NA	7 d after I or II dose (mean value)	NA	NA
	4 PF	NA	NA	10 d after I dose, 6.5 d after II dose (mean values)	NA	NA
(99)	1 MMP	NA	NA	7, 14, and 18 d after I, II, or III dose, respectively (mean values)	NA	NA
(83)	3 MMP	NA	NA	Within 7 d from I to II dose	NA	NA
(100)	79 NS AIBDs	NA	NA	30.4% after I dose, 45.6% after II dose; 24.1% after both	NA	NA

AIBD, autoimmune bullous disease; AZ, Astrazeneca vaccine; AZA, azathioprine; BP, bullous pemphigoid; CV, CoronaVac inactivated vaccine; d, days; DCN, doxycycline; Ig, immunoglobulins; MMF, mycophenolate mofetil; MMP, mucous membrane pemphigoid; Mod, Moderna vaccine; MTX, metothrexate; NA, not available; NS, not specified; P, Pfizer vaccine; pts, patients; PV, pemphigus vulgaris; RTX, rituximab; SCS, systemic corticosteroids; TCS, topical corticosteroids; y, years; Pts, patients.

## COVID-19 vaccination in AIBD patients

Kasperkiewicz and Woodley reported that vaccination did not negatively affect the clinical course of AIBDs in most patients. Only about 6% of post-SARS-CoV-2-vaccinal cases presented clinically with *de novo* AIBDs, and 10% had a flare or worsening of pre-existing AIBDs usually well controlled with standard immune-suppressive treatment (95). However, the fact that vaccination could be less effective during immunosuppressive therapy should be considered. Despite the ongoing treatment with oral corticosteroid, all five AIBD patients analyzed by Damiani et al. developed IgG antibodies against SARS-CoV-2 (> 150 UI) 1 month after receiving the second dose (96). In this context, to achieve maximum efficacy and protection by vaccination, patients should be preferably vaccinated during disease remission or at least under low immunosuppression condition (101, 102). Nonetheless, lowering the corticosteroid dosage before or during the vaccination is not advisable due to the risk of exacerbations (15). Different considerations should be made in case of RTX therapy, especially in unvaccinated patients. B cell-depleting treatments have the most prominent effect on anti-SARS-CoV-2 IgG neutralization titers (103). Specifically, RTX-treated patients have failed to mount any humoral anti-SARS-CoV-2 response, even with a 4–12-month interval between RTX infusion and vaccination (104). Thus, a panel of experts advises that in these patients, vaccination should be performed either before therapy initiation or 4–6 months following RTX infusion (105).

## Conclusion

In COVID-19 and AIBD research area further well-designed investigations are needed. In fact, there are only two studies on large cohorts with standardized findings on prevalence and disease course and very few studies on RTX treatment and COVID-19 disease. In particular, most of the studies do not consider the time between the last RTX treatment and COVID-19, that is the most important parameter to evaluate B-cell population at the time of SARS-CoV-2 infection. Moreover, few studies compare the impact of COVID-19 infection in vaccinated and not-vaccinated patients so far. However, despite of these limitations some important advances can be underlined.

The risks of COVID-19 infection and its severe course were comparable in AIBD patients and in the general population, except for RTX-treated patients. Of note, among COVID-19-infected BP patients the mortality rate was higher than in general population, while for pemphigus data were inconclusive. Thus, AIBD patients during the COVID-19 pandemic, especially if unvaccinated, should be subjected to risk-benefit assessments when considering RTX as a therapeutic option. The causal relationship between vaccination and AIBD onset, relapse or exacerbation has not been demonstrated so far. It could be speculated that vaccination may induce an autoimmune response in genetically predisposed persons by stimulation of pre-existent and sub-clinical autoreactivity. Additional investigations on this controversial topic should be performed. Of course, the risk of these rare adverse events should not discourage vaccination itself, although it should be performed during disease remission or at least while under low immunosuppression.

Finally, the impact of the pandemic on patients and especially on fragile ones has been heavy, but the precious acquired knowledge could also be used in possible, albeit undesirable, future pandemic scenarios.

## Author contributions

AP and GD designed the manuscript. AP, JS, FrM, FeM, and GD wrote the manuscript. GD supervised the work. All authors contributed to the preparation of the manuscript and approved the submitted version.

## References

[1] World Health Organization [WHO]. *COVID-19.* (2022). Available online at: https://covid19.who.int/ (accessed November 28, 2022).

[2] Mahajan S Caraballo C Li SX Dong Y Chen L Huston SK SARS-CoV-2 infection hospitalization rate and infection fatality rate among the non-congregate population in connecticut. *Am J Med.* (2021) 134:812–6.e2. 10.1016/j.amjmed.2021.01.020 33617808 PMC7895685

[3] Petersen E Koopmans M Go U Hamer DH Petrosillo N Castelli F Comparing SARS-CoV-2 with SARS-CoV and influenza pandemics. *Lancet Infect Dis.* (2020) 20:e238–44. 10.1016/S1473-3099(20)30484-9 32628905 PMC7333991

[4] Williamson EJ Walker AJ Bhaskaran K Bacon S Bates C Morton CE Factors associated with COVID-19-related death using OpenSAFELY. *Nature.* (2020) 584:430–6. 10.1038/s41586-020-2521-4 32640463 PMC7611074

[5] Grasselli G Zangrillo A Zanella A Antonelli M Cabrini L Castelli A Baseline characteristics and outcomes of 1591 patients infected with SARS-CoV-2 admitted to ICUs of the lombardy region, Italy. *JAMA.* (2020) 323:1574–81. 10.1001/jama.2020.5394 32250385 PMC7136855

[6] O’Driscoll M Ribeiro Dos Santos G Wang L Cummings DAT Azman AS. Age-specific mortality and immunity patterns of SARS-CoV-2. *Nature.* (2021) 590:140–5. 10.1038/s41586-020-2918-0 33137809

[7] Karagiannidis C Mostert C Hentschker C Voshaar T Malzahn J Schillinger G Case characteristics, resource use, and outcomes of 10 021 patients with COVID-19 admitted to 920 German hospitals: an observational study. *Lancet Respir Med.* (2020) 8:853–62. 10.1016/S2213-2600(20)30316-7 32735842 PMC7386882

[8] Zhou F Yu T Du R Fan G Liu Y. Clinical course and risk factors for mortality of adult inpatients with COVID-19 in Wuhan, China: a retrospective cohort study. *Lancet.* (2020) 395:1054–62. 10.1016/S0140-6736(20)30566-3 32171076 PMC7270627

[9] Gallo Marin B Aghagoli G Lavine K Yang L Siff EJ Chiang SS Predictors of COVID-19 severity: a literature review. *Rev Med Virol.* (2021) 31:1–10. 10.1002/rmv.2146 32845042 PMC7855377

[10] Shang W Dong J Ren Y Tian M Li W Hu J The value of clinical parameters in predicting the severity of COVID-19. *J Med Virol.* (2020) 92:2188–92. 10.1002/jmv.26031 32436996 PMC7280691

[11] Kasperkiewicz M. COVID-19 outbreak and autoimmune bullous diseases: a systematic review of published cases. *J Am Acad Dermatol.* (2021) 84:563–8. 10.1016/j.jaad.2020.08.012 32781181 PMC7414356

[12] Joly P French Study Group on Auto Immune Bullous Skin Diseases, and the French Network of Rare Diseases in Dermatology [FIMARAD]. Incidence and severity of COVID-19 in patients with autoimmune blistering skin diseases: a nationwide study. *J Am Acad Dermatol.* (2022) 86:494–7. 10.1016/j.jaad.2021.10.034 34715285 PMC8553417

[13] Kridin K Schonmann Y Weinstein O Schmidt E Ludwig RJ Cohen AD The risk of COVID-19 in patients with bullous pemphigoid and pemphigus: a population-based cohort study. *J Am Acad Dermatol.* (2021) 85:79–87. 10.1016/j.jaad.2021.02.087 33744354 PMC7968167

[14] Mahmoudi H Farid AS Nili A Dayani D Tavakolpour S Soori T Characteristics and outcomes of COVID-19 in patients with autoimmune bullous diseases: a retrospective cohort study. *J Am Acad Dermatol.* (2021) 84:1098–100. 10.1016/j.jaad.2020.12.043 33359593 PMC7836213

[15] Kasperkiewicz M Yale M Strong R Zillikens D Woodley DT Recke A COVID-19 pandemic and autoimmune bullous diseases: a cross-sectional study of the International Pemphigus and Pemphigoid Foundation. *J Eur Acad Dermatol Venereol.* (2021) 35:e418–21. 10.1111/jdv.17228 33724560 PMC8250878

[16] Balestri R Rech G Girardelli CR. Occurrence of SARS-CoV-2 during mycophenolate mofetil treatment for pemphigus. *J Eur Acad Dermatol Venereol.* (2020) 34:e435–6. 10.1111/jdv.16578 32359182 PMC7267403

[17] Di Altobrando A Patrizi A Abbenante D Bardazzi F. Should SARS-CoV-2 influence immunosuppressive therapy for autoimmune blistering diseases? *J Eur Acad Dermatol Venereol.* (2020) 34:e295–7. 10.1111/jdv.16491 32302437 PMC7264556

[18] Sinagra JL Vedovelli C Binazzi R Salemme A Moro F Mazzanti C Case report: complete and fast recovery from severe COVID-19 in a pemphigus patient treated with rituximab. *Front Immunol.* (2021) 12:665522. 10.3389/fimmu.2021.665522 33936104 PMC8087171

[19] Carugno A Sena P Raponi F Robustelli Test E Vezzoli P. Patients with bullous skin disease in a high-epidemic COVID-19 area, Bergamo, Italy. *Br J Dermatol.* (2020) 183:589–91. 10.1111/bjd.19266 32479659 PMC7301010

[20] Shahidi-Dadras M Abdollahimajd F Ohadi L Tabary L Araghi F Mozafari N COVID-19 in pemphigus vulgaris patients with previous rituximab therapy: a tele-medicine experience. *J Dermatolog Treat.* (2022) 33:1181–2. 10.1080/09546634.2020.1789041 32594787

[21] Özgen Z Aksoy H Akın Çakıcı O Koku Aksu AE Erdem O Polat AK COVID-19 severity and SARS-Cov-2 vaccine safety in pemphigus patients. *Dermatol Ther.* (2022) 35:e15417. 10.1111/dth.15417 35243732 PMC9111561

[22] Uzuncakmak TK Özkoca D Askin O Kutlubay Z. Can rituximab be used in the treatment of pemphigus vulgaris during the COVID-19 pandemic? *Dermatol Ther.* (2021) 34:e14647. 10.1111/dth.14647 33296557 PMC7883057

[23] Baum S Raviv T Gilboa S Pavlotsky F Barzilai A. Efficacy of repeated courses of rituximab as treatment for pemphigus vulgaris. *Acta Dermatol Venereol.* (2020) 100:adv00286. 10.2340/00015555-3649 32985675 PMC9274918

[24] Breglio KF Sarver MM Hall RP III Marano AL. SARS-CoV-2 infections in patients with autoimmune blistering disorders: a case series and retrospective analysis. *J Am Acad Dermatol Int.* (2022) 7:38–43. 10.1016/j.jdin.2022.01.003 35098172 PMC8784432

[25] MacKenna B Kennedy NA Mehrkar A Rowan A Galloway J Matthewman J Risk of severe COVID-19 outcomes associated with immune-mediated inflammatory diseases and immune-modifying therapies: a nationwide cohort study in the OpenSAFELY platform. *Lancet Rheumatol.* (2022) 4:e490–506. 10.1016/S2665-9913(22)00098-4 35698725 PMC9179144

[26] Boekel L Stalman EW Wieske L Hooijberg F van Dam KPJ. Breakthrough SARS-CoV-2 infections with the delta (B.1.617.2) variant in vaccinated patients with immune-mediated inflammatory diseases using immunosuppressants: a substudy of two prospective cohort studies. *Lancet Rheumatol.* (2022) 4:e417–29. 10.1016/S2665-9913(22)00102-3 35527808 PMC9054068

[27] Favalli EG Monti S Ingegnoli F Balduzzi S Caporali R Montecucco C Incidence of COVID-19 in patients with rheumatic diseases treated with targeted immunosuppressive drugs: what can we learn from observational data? *Arthritis Rheumatol.* (2020) 72:1600–6. 10.1002/art.41388 32506699 PMC7300434

[28] Michelena X Borrell H López-Corbeto M López-Lasanta M Moreno E Pascual-Pastor M Incidence of COVID-19 in a cohort of adult and paediatric patients with rheumatic diseases treated with targeted biologic and synthetic disease-modifying anti-rheumatic drugs. *Semin Arthritis Rheum.* (2020) 50:564–70. 10.1016/j.semarthrit.2020.05.001 32425260 PMC7229730

[29] Moghadam FS Kianfar N Dasdar S Samii R Farimani Z Azar PM Adverse outcome and severity of COVID-19 in patients with autoimmune bullous diseases: a historical cohort study. *Dermatol Ther.* (2022) 35:e15672. 10.1111/dth.15672 35768959 PMC9349909

[30] Kow CS Hasan SS. Use of rituximab and the risk of adverse clinical outcomes in COVID-19 patients with systemic rheumatic disease. *Rheumatol Int.* (2020) 40:2117–8. 10.1007/s00296-020-04715-0 33044704 PMC7548404

[31] Guilpain P Le Bihan C Foulongne V Taourel P Pansu N Maria ATJ Response to: ‘Severe COVID-19 associated pneumonia in 3 patients with systemic sclerosis treated with rituximab’ by Avouac et al. *Ann Rheum Dis.* (2021) 80:e38. 10.1136/annrheumdis-2020-217955 32503848

[32] Guilpain P Le Bihan C Foulongne V Taourel P Pansu N Maria ATJ Rituximab for granulomatosis with polyangiitis in the pandemic of COVID-19: lessons from a case with severe pneumonia. *Ann Rheum Dis.* (2021) 80:e10. 10.1136/annrheumdis-2020-217549 32312768

[33] Loarce-Martos J Garcia-Fernandez A Lopez-Gutierrez F Garcia-Garcia V Calvo-Sanz L. High rates of severe disease and death due to SARS-CoV-2 infection in rheumatic disease patients treated with rituximab: a descriptive study. *Rheumatol Int.* (2020) 40:2015–21. 10.1007/s00296-020-04699-x 32945944 PMC7499013

[34] Rondaan C Furer V Heijstek MW Agmon-Levin N Bijl M Breedveld FC Efficacy, immunogenicity and safety of vaccination in adult patients with autoimmune inflammatory rheumatic diseases: a systematic literature review for the 2019 update of EULAR recommendations. *RMD Open.* (2019) 5:e001035. 10.1136/rmdopen-2019-001035 31565247 PMC6744079

[35] Strangfeld A Schafer M Gianfrancesco MA Lawson-Tovey S Liew JW Ljung L Factors associated with COVID-19-related death in people with rheumatic diseases: results from the COVID-19 global rheumatology alliance physician-reported registry. *Ann Rheum Dis.* (2021) 80:930–42. 10.1136/annrheumdis-2020-219498 33504483 PMC7843211

[36] Hwang E Tomayko MM. COVID-19 outcomes in patients with autoimmune blistering disease. *Br J Dermatol.* (2021) 185:1048–50. 10.1111/bjd.20571 34107059 PMC8239772

[37] Di Lillo DJ Hamaguchi Y Ueda Y Yang K Uchida J Haas KM Maintenance of long-lived plasma cells and serological memory despite mature and memory B cell depletion during CD20 immunotherapy in mice. *J Immunol.* (2008) 180:361–71. 10.4049/jimmunol.180.1.361 18097037

[38] Dotan A Muller S Kanduc D David P Halpert G Shoenfeld Y The SARS-CoV-2 as an instrumental trigger Of autoimmunity. *Autoimmun Rev.* (2021) 20:102792. 10.1016/j.autrev.2021.102792 33610751 PMC7892316

[39] De Medeiros VLS Monteiro-Neto AU França DDT Castelo Branco R de Miranda Coelho EO Takano DM Pemphigus vulgaris after COVID-19: a case of induced autoimmunity. *SN Compr Clin Med.* (2021) 3:1768–72. 10.1007/s42399-021-00971-8 34075351 PMC8159521

[40] Ghalamkarpour F Pourani MR. Aggressive course of pemphigus vulgaris following COVID-19 infection. *Dermatol Ther.* (2020) 33:e14398. 10.1111/dth.14398 33040414 PMC7646050

[41] Solimani F Mansour Y Didona D Dilling A Ghoreschi K Meier K Development of severe pemphigus vulgaris following SARS-CoV-2 vaccination with BNT162b2. *J Eur Acad Dermatol Venereol.* (2021) 35:e649–51. 10.1111/jdv.17480 34169588 PMC8447452

[42] Tomayko M Damsky W Fathy R McMahon DE Turner N Valentin MN Subepidermal blistering eruptions, including bullous pemphigoid, following COVID-19 vaccination. *J Allergy Clin Immunol.* (2021) 148:750–1. 10.1016/j.jaci.2021.06.026 34275656 PMC8280592

[43] Larson V Seidenberg R Caplan A Brinster NK Meehan SA Kim RH Clinical and histopathological spectrum of delayed adverse cutaneous reactions following COVID-19 vaccination. *J Cutan Pathol.* (2022) 49:34–41. 10.1111/cup.14104 34292611 PMC8444807

[44] Coto-Segura P Fernández-Prada M Mir-Bonafé M García-García B González-Iglesias I Alonso-Penanes P Vesiculobullous skin reactions induced by COVID-19 mRNA vaccine: report of four cases and review of the literature. *Clin Exp Dermatol.* (2022) 47:141–3. 10.1111/ced.14835 34236711 PMC8444733

[45] McMahon DE Kovarik CL Damsky W Rosenbach M Lipoff JB Tyagi A Clinical and pathologic correlation of cutaneous COVID-19 vaccine reactions including V-REPP: a registry-based study. *J Am Acad Dermatol.* (2022) 86:113–21. 10.1016/j.jaad.2021.09.002 34517079 PMC8431833

[46] Nakamura K Kosano M Sakai Y Saito N Takazawa Y Omodaka T Case of bullous pemphigoid following coronavirus disease 2019 vaccination. *J Dermatol.* (2021) 48:e606–7. 10.1111/1346-8138.16170 34545973 PMC8652433

[47] Agharbi FZ Eljazouly M Basri G Faik M Benkirane A Albouzidi A Bullous pemphigoid induced by the AstraZeneca COVID-19 vaccine. *Ann Dermatol Venereol.* (2022) 149:56–7. 10.1016/j.annder.2021.07.008 34686374 PMC8481089

[48] Young J Mercieca L Ceci M Pisani D Betts A Boffa MJ A case of bullous pemphigoid after the SARS-CoV-2 mRNA vaccine. *J Eur Acad Dermatol Venereol.* (2022) 36:e13–6. 10.1111/jdv.17676 34547137 PMC8661451

[49] Thongprasom K Pengpis N Phattarataratip E Samaranayake L. Oral pemphigus after COVID-19 vaccination. *Oral Dis.* (2022) 28(Suppl. 2):2597–8. 10.1111/odi.14034 34582621 PMC8662146

[50] Schmidt V Blum R Möhrenschlager M. Biphasic bullous pemphigoid starting after first dose and boosted by second dose of mRNA-1273 vaccine in an 84-year-old female with polymorbidity and polypharmacy. *J Eur Acad Dermatol Venereol.* (2022) 36:e88–90. 10.1111/jdv.17722 34606112

[51] Pérez-López I Moyano-Bueno D Ruiz-Villaverde R. Bullous pemphigoid and COVID-19 vaccine. *Med Clin (Engl Ed).* (2021) 157:e333–4. 10.1016/j.medcle.2021.05.004 34697598 PMC8529265

[52] Hali F Kerouach A Alatawna H Chiheb S Lakhdar H. Linear IgA bullous dermatosis following Oxford AstraZeneca COVID-19 vaccine. *Clin Exp Dermatol.* (2022) 47:611–3. 10.1111/ced.15007 34762342 PMC8652795

[53] Lua ACY Ong FLL Choo KJL Yeo YW Oh CC. An unusual presentation of pemphigus foliaceus following COVID-19 vaccination. *Australas J Dermatol.* (2022) 63:128–30. 10.1111/ajd.13755 34817063

[54] Dell’Antonia M Anedda S Usai F Atzori L Ferreli C. Bullous pemphigoid triggered by COVID-19 vaccine: rapid resolution with corticosteroid therapy. *Dermatol Ther.* (2022) 35:e15208. 10.1111/dth.15208 34786801 PMC8646458

[55] Hatami P Balighi K Nicknam Asl H Aryanian Z. COVID vaccination in patients under treatment with rituximab: a presentation of two cases from Iran and a review of the current knowledge with a specific focus on pemphigus. *Dermatol Ther.* (2022) 35:e15216. 10.1111/dth.15216 34811862 PMC9011959

[56] Koutlas IG Camara R Argyris PP Davis MDP Miller DD. Development of pemphigus vulgaris after the second dose of the mRNA-1273 SARS-Cov-2 vaccine. *Oral Dis.* (2022) 28(Suppl. 2):2612–3. 10.1111/odi.14089 34825752

[57] Bostan E Yel B Akdogan N Gokoz O. New-onset bullous pemphigoid after inactivated COVID-19 vaccine: synergistic effect of the COVID-19 vaccine and vildagliptin. *Dermatol Ther.* (2022) 35:e15241. 10.1111/dth.15241 34854184

[58] Knechtl GV Seyed Jafari SM Berger T Rammlmair A Feldmeyer L Borradori L Development of pemphigus vulgaris following mRNA SARS-CoV-19 BNT162b2 vaccination in an 89-year-old patient. *J Eur Acad Dermatol Venereol.* (2022) 36:e251–3. 10.1111/jdv.17868 34897817

[59] Pauluzzi M Stinco G Errichetti E. Bullous pemphigoid in a young male after COVID-19 mRNA vaccine: a report and brief literature review. *J Eur Acad Dermatol Venereol.* (2022) 36:e257–9. 10.1111/jdv.17891 34928518

[60] Gambichler T Hamdani N Budde H Sieme M Skrygan M Scholl L Bullous pemphigoid after SARS-CoV-2 vaccination: spike-protein-directed immunofluorescence confocal microscopy and T-cell-receptor studies. *Br J Dermatol.* (2022) 186:728–31. 10.1111/bjd.20890 34773638 PMC8653321

[61] Afacan E Edek YC Ýlter N Gülekon A. Can COVID-19 vaccines cause or exacerbate bullous pemphigoid? A report of seven cases from one center. *Int J Dermatol.* (2022) 61:626–7. 10.1111/ijd.16086 35080255

[62] Hung WK Chi CC. Incident bullous pemphigoid in a psoriatic patient following mRNA-1273 SARS-CoV-2 vaccination. *J Eur Acad Dermatol Venereol.* (2022) 36:e407–9. 10.1111/jdv.17955 35073431

[63] Koumaki D Krueger-Krasagakis SE Papadakis M Katoulis AC Gkiaouraki I Zografaki K Psoriasis flare-up after AZD1222 and BNT162b2 COVID-19 mRNA vaccines: report of twelve cases from a single centre. *J Eur Acad Dermatol Venereol.* (2022) 36:e411–5. 10.1111/jdv.17965 35075691

[64] Yıldırıcı S Yaylı S Demirkesen C Vural S. New onset of pemphigus foliaceus following BNT162b2 vaccine. *Dermatol Ther.* (2022) 35:e15381. 10.1111/dth.15381 35170156 PMC9111864

[65] Maronese CA Caproni M Moltrasio C Genovese G Vezzoli P Sena P Bullous pemphigoid associated with COVID-19 vaccines: an Italian multicentre study. *Front Med (Lausanne).* (2022) 9:841506. 10.3389/fmed.2022.841506 35295599 PMC8918943

[66] Alshammari F Abuzied Y Korairi A Alajlan M Alzomia M AlSheef M Bullous pemphigoid after second dose of mRNA- (Pfizer-BioNTech) COVID-19 vaccine: a case report. *Ann Med Surg (Lond).* (2022) 75:103420. 10.1016/j.amsu.2022.103420 35251600 PMC8885466

[67] Calabria E Canfora F Mascolo M Varricchio S Mignogna MD Adamo D Autoimmune mucocutaneous blistering diseases after SARS-Cov-2 vaccination: a case report of pemphigus vulgaris and a literature review. *Pathol Res Pract.* (2022) 232:153834. 10.1016/j.prp.2022.153834 35278817 PMC8896864

[68] Hali F Sr. Araqi L Jr. Marnissi F Meftah A Chiheb S. Autoimmune bullous dermatosis following COVID-19 vaccination: a series of five cases. *Cureus.* (2022) 14:e23127. 10.7759/cureus.23127 35425676 PMC9004698

[69] Akoglu G. Pemphigus vulgaris after SARS-CoV-2 vaccination: a case with new-onset and two cases with severe aggravation. *Dermatol Ther.* (2022) 35:e15396. 10.1111/dth.15396 35187768 PMC9111794

[70] Saffarian Z Samii R Ghanadan A Vahidnezhad H. De novo severe pemphigus vulgaris following SARS-CoV-2 vaccination with BBIBP-CorV. *Dermatol Ther.* (2022) 35:e15448. 10.1111/dth.15448 35289040 PMC9111647

[71] Singh A Bharadwaj SJ Chirayath AG Ganguly S. Development of severe pemphigus vulgaris following ChAdOx1 nCoV-19 vaccination and review of literature. *J Cosmet Dermatol.* (2022) 21:2311–4. 10.1111/jocd.14945 35348281 PMC9115051

[72] Maronese CA Di Zenzo G Genovese G Barei F Monestier A Pira A Reply to “New-onset bullous pemphigoid after inactivated COVID-19 vaccine: synergistic effect of the COVID-19 vaccine and vildagliptin”. *Dermatol Ther.* (2022) 35:e15496. 10.1111/dth.15496 35388592 PMC9111836

[73] Desai AD Shah R Haroon A Wassef C. Bullous pemphigoid following the moderna mRNA-1273 vaccine. *Cureus.* (2022) 14:e24126. 10.7759/cureus.24126 35573488 PMC9106564

[74] Han J Russo G Stratman S Psomadakis CE Rigo R Owji S Toxic epidermal necrolysis-like linear IgA bullous dermatosis after third Moderna COVID-19 vaccine in the setting of oral terbinafine. *J Am Acad Dermatol Case Rep.* (2022) 24:101–4. 10.1016/j.jdcr.2022.04.021 35571457 PMC9077902

[75] Bardazzi F Carpanese MA Abbenante D Filippi F Sacchelli L Loi C New-onset bullous pemphigoid and flare of pre-existing bullous pemphigoid after the third dose of the COVID-19 vaccine. *Dermatol Ther.* (2022) 35:e15555. 10.1111/dth.15555 35510556 PMC9347770

[76] Bailly-Caillé B Jouen F Dompmartin A Morice C. A case report of anti-P200 pemphigoid following COVID-19 vaccination. *JAAD Case Rep.* (2022) 23:83–6. 10.1016/j.jdcr.2022.03.011 35308065 PMC8923033

[77] Zhang Y Lang X Guo S He H Cui H. Bullous pemphigoid after inactivated COVID-19 vaccination: case report. *Dermatol Ther.* (2022) 35:e15595. 10.1111/dth.15595 35608483 PMC9347905

[78] Shanshal M. Dyshidrosiform bullous pemphigoid triggered by COVID-19 vaccination. *Cureus.* (2022) 14:e26383. 10.7759/cureus.26383 35911257 PMC9329707

[79] Shakoei S Kalantari Y Nasimi M Tootoonchi N Ansari MS Razav Z Cutaneous manifestations following COVID-19 vaccination: a report of 25 cases. *Dermatol Ther.* (2022) 35:e15651. 10.1111/dth.15651 35716105 PMC9349410

[80] Gui H Young PA So JY Pol-Rodriguez M Rieger KE Lewis MA New-onset pemphigus vegetans and pemphigus foliaceus after SARS-CoV-2 vaccination: a report of 2 cases. *JAAD Case Rep.* (2022) 27:94–8. 10.1016/j.jdcr.2022.07.002 35845348 PMC9270232

[81] Avallone G Cavallo F Astrua C Caldarola G Conforti C De Simone C Cutaneous adverse reactions following SARS-CoV-2 vaccine booster dose: a real-life multicentre experience. *J Eur Acad Dermatol Venereol.* (2022) 36:e876–9. 10.1111/jdv.18386 35771093 PMC9349823

[82] Alelq NA Kubieniec ME French LE Prinz JC. Influence of COVID-19 vaccination on immune-mediated skin diseases. *J Eur Acad Dermatol Venereol.* (2022) 36:e965–8. 10.1111/jdv.18388 35771095 PMC9349455

[83] Kianfar N Dasdar S Farid AS Balighi K Mahmoudi H Daneshpazhooh M Exacerbation of autoimmune bullous diseases after severe acute respiratory syndrome coronavirus 2 vaccination: is there any association? *Front Med (Lausanne).* (2022) 9:957169. 10.3389/fmed.2022.957169 35928293 PMC9344059

[84] Daines B Madigan LM Vitale PA Khalighi M Innes M. A new eruption of bullous pemphigoid following mRNA COVID-19 vaccination. *Dermatol Online J.* (2022) 28:11. 10.5070/D328458525 36259862

[85] Corrá A Barei F Genovese G Zussino M Spigariolo CB Mariotti EB Five cases of new-onset pemphigus following vaccinations against coronavirus disease 2019. *J Dermatol.* (2022) 17:10.1111/1346–8138.16554. 10.1111/1346-8138.16554 35975548 PMC9538601

[86] Nahm WJ Juarez M Wu J Kim RH. Eosinophil-rich linear IgA bullous dermatosis induced by mRNA COVID-19 booster vaccine. *J Cutan Pathol.* (2022) 50:24–8. 10.1111/cup.14305 35922892 PMC9538274

[87] Pouran M Bidari-Zerehpoosh F Ayatollahi A Robati RM. New onset of pemphigus foliaceus following BBIBP COVID-19 vaccine. *Dermatol Ther.* (2022) 35:e15816. 10.1111/dth.15816 36093743 PMC9538718

[88] Al Maashari RS Al Mahmood Noaf S Tausif S. Vesiculo-bullous eruption following COVID vaccination. *Saudi J Med.* (2022) 7:501–4.

[89] Chao YC Liu KL. New-onset bullous pemphigoid triggered by AstraZeneca COVID-19 vaccine. *Dermatol Sin.* (2022) 40:245–6.

[90] Wan V Chen D Shiau CJ Jung GW. Association between COVID-19 vaccination and bullous pemphigoid–a case series and literature review. *SAGE Open Med Case Rep.* (2022) 10:2050313X221131868. 10.1177/2050313X221131868 36274858 PMC9580082

[91] Baffa ME Maglie R Montefusco F Pipitò C Senatore S. Severe bullous pemphigoid following COVID-19 vaccination resistant to rituximab and successfully treated with dupilumab. *J Eur Acad Dermatol Venereol.* (2022): [Epub ahead of print]. 10.1111/jdv.18673 36268775

[92] Birabaharan M Kaelber DC Orme CM Paravar T Karris MY. Evaluating risk of bullous 115 pemphigoid after mRNA COVID-19 vaccination. *Br J Dermatol.* (2022) 187:271–3. 10.1111/bjd.21240 35279833 PMC9111668

[93] Korang K Ghohestani R Krieg T Uitto J Hunzelmann N. Exacerbation of pemphigus foliaceus after tetanus vaccination accompanied by synthesis of auto-antibodies against paraneoplastic pemphigus antigens. *Acta Dermato Venereol.* (2002) 82:482–3. 10.1080/000155502762064755 12575868

[94] De Simone C Caldarola G D’Agostino M Zampetti A Amerio P. Exacerbation of pemphigus after influenza vaccination. *Clin Exp Dermatol.* (2008) 33:718–20. 10.1111/j.1365-2230.2008.02835.x 18681883

[95] Kasperkiewicz M Woodley DT. Association between vaccination and immunobullous disorders: a brief, updated systematic review with focus on COVID-19. *J Eur Acad Dermatol Venereol.* (2022) 36:e498–500. 10.1111/jdv.18030 35220622 PMC9114988

[96] Damiani G Pacifico A Pelloni F Iorizzo M. The first dose of COVID-19 vaccine may trigger pemphigus and bullous pemphigoid flares: is the second dose therefore contraindicated? *J Eur Acad Dermatol Venereol.* (2021) 35:e645–7. 10.1111/jdv.17472 34169578 PMC8447358

[97] Saleh MA Saleh NA. Pemphigus vulgaris relapse during the coronavirus disease pandemic. *Dermatol Ther.* (2022) 35:e15354. 10.1111/dth.15354 35108427

[98] Ong SK Darji K Chaudhry SB. Severe flare of pemphigus vulgaris after first dose of COVID-19 vaccine. *J Am Acad Dermatol Case Rep.* (2022) 22:50–2. 10.1016/j.jdcr.2022.01.027 35169602 PMC8830178

[99] Sprow G Afarideh M Dan J Feng R Keyes E Grinnell M Autoimmune skin disease exacerbations following COVID-19 vaccination. *Front Immunol.* (2022) 13:899526. 10.3389/fimmu.2022.899526 35693768 PMC9186119

[100] Kasperkiewicz M Strong R Mead K Yale M Zillikens D Woodley DT COVID-19 vaccine acceptance and hesitancy in patients with immunobullous diseases: a cross-sectional study of the international pemphigus and pemphigoid foundation. *Br J Dermatol.* (2022) 186:737–9. 10.1111/bjd.20906 34842282

[101] Kasperkiewicz M Schmidt E Fairley JA Joly P Payne AS Yale ML Expert recommendations for the management of autoimmune bullous diseases during the COVID-19 pandemic. *J Eur Acad Dermatol Venereol.* (2020) 34:e302–3. 10.1111/jdv.16525 32333823 PMC7267551

[102] Seree-Aphinan C Chanprapaph K Rattanakaemakorn P Setthaudom C Suangtamai T Pomsoong C Inactivated COVID-19 vaccine induces a low humoral immune response in a subset of dermatological patients receiving immunosuppressants. *Front Med.* (2021) 8:769845. 10.3389/fmed.2021.769845 34957149 PMC8692273

[103] Deepak P Kim W Paley MA Yang M Carvidi AB El-Qunni AA Glucocorticoids and B cell depleting agents substantially impair immunogenicity of mRNA vaccines to SARS-CoV-2. *medRxiv [Preprint].* (2021): 10.1101/2021.04.05.21254656 33851176 PMC8043473

[104] Bonelli MM Mrak D Perkmann T Haslacher H Aletaha D. SARS-CoV-2 vaccination in rituximab-treated patients: evidence for impaired humoral but inducible cellular immune response. *Ann Rheum Dis.* (2021) 80:1355–6. 10.1136/annrheumdis-2021-220408 33958323

[105] European Reference Network [ERN] Skin. *Vaccination Advices.* (2022) Available online at: https://ern-skin.eu/vaccination-advices/# (accessed November 30, 2022).

