# Supplementary material for: Autoimmune bullous diseases during COVID-19 pandemic: 2022 update on rituximab and vaccine
Source: Front Med (Lausanne). 2023 Jan 19;10:1112823. doi: 10.3389/fmed.2023.1112823 (PMC9893122; doi:10.3389/fmed.2023.1112823)
Supplement: Supplementary file 1 [file Table_1.pdf]

Table 1 supplementary. Demographics, vaccines, clinical and immunopathological features of patients AIBD associated with COVID-19 vaccination

Ref.	Pts	Sex, age (y)	Vaccine	Latency to Lesions	Hist[a]	DIF[b]	IIF[c]	ELISA BP180/BP230	ELISA Dsg1/Dsg3	Therapy	Outcome
**42**	12 BP	F, 97	P	2d after II dose	pos	pos	pos	pos/pos	-	TCS, DCN, NAM	Improvement
		M, 75	P	10d after II dose	pos	pos	ND	pos/ND	-	SCS, TCS, DCN, NAM	Improvement
		M, 64	P	14d after II dose	pos	pos	neg	pos/pos	-	TCS	Improvement
		M, 82	P	1d after II dose	pos	pos	pos	neg/neg	-	TCS	Remission
		F, 95	P	5d after I dose	pos	pos	pos	neg/neg	-	TCS, DCN, NAM	Remission
		M, 87	Mod	21d after II dose	pos	pos	ND	pos/pos	-	SCS, DCN, NAM	Ongoing at w15
		F, 42	Mod	3d after II dose	pos	pos	ND	pos/pos	-	TCS, SCS	Ongoing at w4
		M, 85	P	5d after I dose	pos	pos	ND	ND	-	OCS	Ongoing at w9
		F, 83	Mod	8d after I dose	pos	neg	neg	neg/neg	-	TCS, SCS	Ongoing at mo2
		F, 66	P	7d after I dose	pos	neg	neg	neg/neg	-	TCS, SCS	Remission
		F, 70	Mod	9d after I dose	pos	neg	neg	ND	-	SCS	Remission
		F, 83	P	7d after II dose	pos	ND	ND	ND	-	SCS, TCS, DCN, NAM	Ongoing at w6
**43**	2 BP	M, 76	P	21d after I dose	NA	pos	pos	NA	-	TCS, SCS, AH, DCN, NAM	Improvement
		M, 84	Mod	14d after II dose	neg	pos	NA	NA	-	TCS, SCS	Improvement
**44**	3 BP	M, 86	P	18d after II dose	pos	pos	NA	NA	-	TCS, SCS	Improvement
		M, 85	P	8d after II dose	pos	pos	NA	NA	-	TCS, SCS, AH	Improvement
		M, 84	P	7d after II dose	pos	pos	NA	NA	-	TCS, SCS	Improvement
**45**	12 BP	NA	P 64%, Mod 36%	NA	pos	pos	NA	pos/NA	-	NA	NA
		NA		NA	pos	pos	NA	NA	-	NA	NA
		NA		NA	pos	pos	NA	NA	-	NA	NA
		NA		NA	pos	pos	NA	NA	-	NA	NA
		NA		NA	pos	pos	NA	NA	-	NA	NA
		NA		NA	pos	pos	NA	NA	-	NA	NA

		NA		NA	pos	neg	NA	NA	-	NA	NA
		NA		NA	pos	neg	NA	NA	-	NA	NA
		NA		NA	pos	NA	NA	NA	-	NA	NA
		NA		NA	pos	NA	NA	NA	-	NA	NA
		NA		NA	pos	NA	NA	NA	-	NA	NA
		NA		NA	pos	NA	NA	NA	-	NA	NA
**46**	1 BP	F, 83	P	3d after II dose	pos	pos	NA	pos/NA	-	SCS, Ig	Improvement
**47**	1 BP	M, 77	AZ	1d after I dose	pos	pos	pos	NA	-	TCS, DCN	Improvement
**48**	1 BP	M, 68	P	3d after I dose	pos	pos	NA	NA	-	TCS	Remission
**50**	1 BP	F, 84	Mod	few d after I dose	pos	ND	ND	pos/pos	-	NA	NA
**51**	1 BP	F, 78	P	3d after I dose	neg	pos	pos	neg/neg	-	SCS	Ongoing at w3
**54**	1 BP	M, 83	P	7d after I dose	pos	pos	NA	NA	-	TCS	Complete remission
**57**	1 BP	M, 67	in. vacc.	35d after I dose	pos	pos	NA	NA	-	SCS, OMAL	Improvement
**59**	1 BP	M, 46	P	15d after I dose	pos	pos	NA	pos/NA	-	SCS, AZA	Ongoing at w7
**60**	2 BP	M, 80	P	7d after I dose	pos	pos	pos	pos/pos	-	SCS	Improvement
		M, 89	P	3d after I dose	pos	pos	pos	pos/pos	-	SCS	Improvement
**61**	4 BP	F, 88	CV	30 days after II dose	pos	pos	NA	NA	-	TCS, SCS, MTX	COVID-19 infection while under SCS + MTX
		F, 82	P	14 days after III dose	pos	pos	NA	NA	-	TCS, SCS, DAP	Improvement
		M, 65	P	14 days after III dose	pos	pos	NA	NA	-	TCS, DCN	Improvement
		F, 82	CV	14 days after II dose	pos	pos	NA	NA	-	TCS, SCS	Improvement
**62**	1 BP	M, 39	Mod	30d after I dose	NA	pos	pos	NA	-	SCS	Improvement
**63**	1 BP	M, 61	AZ	60d after I dose	NA	NA	NA	NA	-	SCS	Improvement
**65**	21 BP	F, 84	P	25d after I dose	pos	pos	pos	pos/neg	-	TCS, SCS, DCN	Improvement
		M, 83	P	32d after I dose	pos	pos	pos	pos/pos	-	TCS, SCS, DCN	Improvement
		F, 56	Mod	7d after I dose	pos	neg	pos	pos/pos	-	TCS, DCN	Improvement
		M, 79	P	4d after I dose	pos	pos	pos	pos/neg	-	TCS, DCN	Improvement
		M, 86	P	37d after I dose	pos	pos	pos	pos/neg	-	TCS	Improvement

		M, 91	P	28d after I dose	pos	pos	pos	neg/neg	-	TCS, SCS	Improvement
		M, 86	P	36d after I dose	pos	pos	NA	NA	-	TCS, SCS, DCN	Improvement
		F, 84	Mod	7d after I dose	pos	pos	pos	pos/neg	-	TCS, SCS, DCN	Improvement
		M, 84	P	23d after I dose	pos	pos	NA	neg/neg	-	SCS	Improvement
		F, 82	P	34d after I dose	pos	pos	NA	neg/neg	-	SCS	Improvement
		M, 76	P	34d after I dose	pos	pos	NA	neg/neg	-	SCS	NA
		M, 78	P	4d after I dose	pos	NA	pos	pos/pos	-	TCS	Improvement
		F, 90	P	28d after I dose	pos	pos	pos	pos/neg	-	TCS, SCS	Improvement
		M, 90	P	64d after I dose	pos	pos	neg	neg/neg	-	SCS	Improvement
		M, 72	P	16d after I dose	pos	pos	neg	pos/neg	-	TCS, SCS, MTX	Improvement
		M, 80	P	6d after I dose	pos	pos	pos	NA	-	TCS, SCS	Improvement
		F, 77	AZ	3d after I dose	pos	pos	pos	pos/pos	-	MTX	Improvement
		F, 60	P	75d after I dose	pos	pos	pos	pos/pos	-	SCS	Improvement
		F, 70	P	27d after I dose	pos	pos	pos	neg/neg	-	SCS	Improvement
		F, 72	AZ	7d after I dose	pos	NA	NA	NA	-	SCS, DAP	Improvement
		M, 85	P	27d after I dose	pos	NA	NA	NA	-	SCS	Ongoing at mo3
**66**	1 BP	M, 78	P	1d after II dose	NA	pos	NA	NA	-	SCS, ABX	Improvement
**68**	3 BP	M, 51	AZ	7d after II dose	pos	pos	pos	NA	-	SCS	Complete remission
		F, 54	AZ	3d after I dose	pos	pos	pos	NA	-	TCS	Improvement
		M, 68	AZ	14d after I dose	pos	pos	NA	NA	-	SCS	Improvement
**72**	3 BP	M, 67	P	21-28d after I dose	pos	pos	pos	pos/pos	-	TCS, DCN	Improvement
		F, 84	P	28d after I dose	NA	NA	NA	pos*/neg	-	TCS, SCS	Improvement
		M, 86	P	14d after I dose	NA	NA	NA	pos/neg	-	TCS, SCS	Improvement
**73**	1 BP	F, 73	Mod	1d after II dose	pos	pos	NA	NA	-	SCS, MMF	Improvement
**75**	2 BP	F, 76	P	12d after III dose	pos	pos	NA	pos/pos	-	TCS, SCS	Complete remission
		M, 79	P	9d after III dose	pos	pos	NA	pos/pos	-	TCS, SCS, NAM	Complete remission
**77**	2 BP	M, 23	CV	1d after III dose	pos	pos	pos	pos/pos	-	SCS	Disease control
		M, 81	CV	15d after III dose	pos	pos	NA	pos/neg	-	SCS, GG	Improvement

**78**	1 BP	F, 90	P	7d after I dose	pos	pos	pos	NA	-	NA	NA
**79**	2 BP	F, 85	CV	20d after I dose	NA	NA	NA	NA	-	TCS, DCN	Improvement
		M, 91	CV	19d after I dose	NA	NA	NA	NA	-	TCS, RTX	Improvement
**81**	1 BP	M, 72	Mod	20d after III dose	pos	pos	NA	NA	-	NA	NA
**82**	1 BP	F, 81	P	14d after I dose	pos	pos	NA	pos/pos	-	TCS, SCS	Improvement
**83**	3 BP	3 pts	NA	NA	NA	NA	NA	NA	-	NA	NA
**84**	1 BP	M, 70	P	1d after II dose	pos	pos	pos	pos/neg	-	TCS, SCS, ABX, MTX	Improvement
**88**	1 BP	M, 30	CV	3d after I dose	pos	pos	ND	ND	-	SCS, DCN	Improvement
**89**	1 BP	F, 49	AZ	5d after II dose	pos	pos	pos	NA	-	TCS, SCS, AZA	Improvement
**90**	2 BP	F, 50	P	14d after II dose	pos	pos	NA	NA	-	SCS, MTX	Disease control
		M, 82	P	10d after I dose	pos	pos	NA	NA	-	TCS	Remission
**91**	1 BP	F, 91	P	10d after II dose	pos	pos	pos	pos/NA	-	TCS, SCS, RTX, Ig, AZA	Remission
**76**	1 p200 pemphigoid	M, 74	Mod	2d after II dose	pos	pos	pos	neg/neg	-	TCC	Remission
**41**	1 PV	F, 40	P	5d after I dose	pos	pos	NA	-	pos/pos	SCS, AZA	Improvement
**49**	1 pemphigus	F, 38	AZ	7d after I dose	pos	pos	NA	-	NA	TCS	Remission
**53**	1 PF	M, 83	P	2d after II dose	pos	pos	pos	-	pos/neg	SCS	Improvement
**55**	1 PV	M, 34	AZ	"few"d after I dose	pos	pos	NA	-	NA	SCS, AZA	NA
**56**	1 PV	M, 60	Mod	7d after II dose	pos	pos	NA	-	neg/neg	SCS, RTX	Remission
**58**	1 PV	M, 89	P	30d after II dose	pos	pos	NA	-	pos/pos	SCS, RTX	Disease control at w10
**64**	1 PF	M, 65	P	21d after I dose	pos	pos	NA	-	pos/neg	SCS, AZA	Improvement
**67**	1 PV	F, 60	P	7d after II dose	pos	pos	NA	-	neg/pos	SCS, RTX	Partial remission
**68**	1 PF	F, 50	P	15d after I dose	pos	pos	pos	-	NA	SCS	Complete remission
	1 PV	F, 58	P	30d after I dose	pos	pos	pos	-	NA	SCS	NA
**69**	1 PV	F, 69	CV	7 days after II dose	NA	NA	NA	-	pos/pos	SCS, MTX	Remission
**70**	1 PV	F, 76	CV	30d after II dose	pos	pos	NA	-	neg/neg	SCS, RTX	Improvement
**71**	1 PV	M, 44	AZ	7d after II dose	pos	NA	NA	-	NA/pos	SCS, ABX, AZA	Improvement
**79**	1 PV	F, 30	CV	16d after I dose	NA	NA	NA	-	NA	SCS, RTX	Improvement
**80**	1 PVeg	M, 25	P	30d after II dose	NA	pos	pos	-	NA	TCS, SCS, MMF	Improvement

	1 PF	F, 67	Mod	14d after II dose	pos	pos	pos	-	pos/neg	TCS, SCS, ABX	Remission
**83**	2 PV	2 pts	NA	NA	NA	NA	NA	-	NA	NA	NA
**85**	3 PV	F, 61	P	3d after III dose	pos	pos	pos	-	pos/pos	SCS	Improvement
		F, 73	P	28d after III dose	pos	pos	pos	-	NA/pos	SCS, RTX	Improvement
		F, 63	AZ	28d after I dose	pos	pos	pos	-	pos/pos	SCS, RTX	Partial remission
	2 PF	M, 80	P	17d after III dose	pos	neg	pos	-	pos/NA	SCS, RTX, MMF	Improvement
		F, 66	P	28d after II dose	pos	pos	neg	-	neg/neg	SCS, MMF	Improvement
**87**	1 PF	M, 75	CV	14d after III dose	pos	pos	NA	-	NA	TCS, RTX	Improvement
**44**	1 LABD	M, 71	P	3d after II dose	pos	pos	NA	-	-	TCS	Improvement
**52**	1 LABD	M, 61	AZ	3d after II dose	pos	pos	pos	neg/neg	neg/neg	SCS	Improvement
**74**	1 LABD	F, 86	Mod	1d after III dose	NA	pos	NA	-	-	SCS, ABX	Remission
**86**	1 LABD	M, 66	Mod	5d after III dose	pos	pos	pos	neg/neg	-	TCS, SCS, DAP	Remission

[a]***Histopathological examination is marked as positive if it's considered consistent with the patient's diagnosis:***

- Bullous pemphigoid: subepidermal blistering and eosinophil-rich infiltrates
- Pemphigus: intraepidermal acantholysis with intact basal layer (tombstone sign) and intraepidermal eosinophils
- LABD: subepidermal blisters with neutrophilic infiltration

[b]***DIF is marked as positive if it's considered consistent with the patient's diagnosis:***

- Bullous pemphigoid: linear deposition of IgG/C3 along the dermo-epidermal junction
- Pemphigus: intraepidermal deposition of IgG/IgA/C3
- LABD: linear deposition of IgA along the dermo-epidermal junction

[c]***IIF is marked as positive if it's considered consistent with the patient's diagnosis:***

- Bullous pemphigoid: linear staining of IgG/C3 along the dermo-epidermal junction; immunoreactants localize to blister roof (95%) on salt split analysis
- Pemphigus: intercellular staining of IgG/IgA
- LABD: linear staining of IgA along the dermo-epidermal junction

*BP180 reactivity detected by immunoblotting on keratinocyte extracts

ABX: antibiotics; *AH:* anti-histamine; *AIBD:* autoimmune bullous disease; *AZ:* Astrazeneca; *AZA:* azathioprine; *BP:* bullous pemphigoid; *CV:* Coronavac; *d:* days; *DAP:* dapsone; *DCN:* doxycycline; *DIF:* direct immunofluorescence; *Ig:* Immunoglobulins; *in. vacc.:* inactivated vaccine; *GG:* gamma globulins *Hist:* Histopathologic examination; *IIF:* indirect immunofluorescence; *LABD:* linear IgA bullous dermatosis; *MMF:* mycophenolate mofetil; *mo:* months; *Mod:* Moderna; *MTX:* methotrexate; *NA:* not available; *NAM:* nicotinamide; *ND:* not done; *neg:* negative; *P:* Pfizer; *PF:* pemphigus foliaceus; *pos:* positive; *pts:* patients; *PV:* pemphigus vulgaris; *PVeg:* pemphigus vegetans; *RTX:* Rituximab; *SCS:* systemic corticosteroids; *TCS:* topical corticosteroids *y:* years; *w:* weeks.
